# A rare case report of relapsed/refractory primary cutaneous diffuse large B-cell lymphoma, leg type, treated with Epcoritamab: results obtained and lesson learned

**DOI:** 10.3389/fonc.2026.1710502

**Published:** 2026-02-10

**Authors:** Alessandro Cafaro, Francesco Malaspina, Pietro Rossi, Costantino Riemma, Jessica Rosa, Eliana Valentina Liardo, Margherita Parolini, Matelda Medri, Paolo Silimbani, Laura Crudi, Carla Masini, Gerardo Musuraca

**Affiliations:** 1 Pharmacy Unit, IRCCS Istituto Romagnolo per lo Studio dei Tumori (IRST) “Dino Amadori”, Meldola, Italy; 2 Hematology Unit, IRCCS Istituto Scientifico Romagnolo per lo Studio dei Tumori (IRST) “Dino Amadori”, Meldola, Italy; 3 Skin Cancer Unit, Istituto Scientifico Romagnolo per lo Studio e la Cura dei Tumori (IRST), Istituto di Ricovero e Cura a Carattere Scientifico (IRCCS), Meldola, Italy

**Keywords:** CD20, Epcoritamab, leg type, primary cutaneous diffuse large B-cell lymphoma, skin lesions

## Abstract

Primary Cutaneous Diffuse Large B-Cell Lymphoma, Leg Type (PCDLBCL-LT), is a rare and aggressive subtype of primary cutaneous B-cell lymphoma, predominantly affecting elderly individuals. The disease presents with nodular or tumorous skin lesions, mainly on the legs, and follows a highly aggressive clinical course, with frequent relapses and poor prognosis. The standard first-line treatment involves Rituximab-based chemoimmunotherapy (R-CHOP), but therapeutic options for relapsed or refractory cases remain limited. We report the case of a 72-year-old male with relapsed/refractory PCDLBCL-LT who achieved a remarkable but short-lived response to Epcoritamab, a novel bispecific antibody targeting CD3 and CD20. After multiple treatment lines, including R-COMP, Tafasitamab-Lenalidomide, and Polatuzumab-Rituximab, the patient received Epcoritamab as fourth-line therapy. Despite an initial clinical response with regression of cutaneous lesions, early relapse occurred, coinciding with loss of CD20 expression, likely due to prolonged Rituximab exposure. This highlights an already described mechanism of resistance in T cell-CD20-directed therapies. To our best knowledge, this is the first reported case of relapsed/refractory PCDLBCL-LT treated with a bispecific antibody targeting CD20 alone, without a previous CAR T.

## 1 Introduction

Primary cutaneous diffuse large B-cell lymphoma, leg type (PCDLBCL-LT), is a rare and aggressive variant of primary cutaneous B-cell lymphomas. This neoplasm predominantly affects elderly individuals, with a median age of onset of 76 years, and has a predilection for the lower limbs, involving the legs in 72% of cases (1).

DLBCL and PCDLBCL are similar in that both are aggressive B-cell lymphomas originating from similar cell types, with PCDLBCL often sharing molecular and genetic features with the activated B-cell (ABC) subtype of systemic DLBCL. However they differ in their primary location and clinical presentation (2).

Clinically, PCDLBCL-LT presents with nodular or tumorous skin lesions of a reddish-violet color, often multiple and primarily located on the legs. The disease follows an aggressive clinical course, with frequent relapses and a tendency for extracutaneous dissemination. Studies have reported a 5-year disease-specific survival rate of 41%, with a particularly poor prognosis in patients with multiple leg lesions (1).

At the genetic level, PCDLBCL-LT is often associated with high expression of the Bcl-2 protein (85% of cases), which inhibits apoptosis and promotes tumor cell survival. Additionally, genetic alterations such as MYC gene translocation, which drives cell proliferation, and TNFAIP3 gene mutations, leading to NF-kB pathway activation, contribute to the disease’s aggressiveness (3–5).

The diagnosis of PCDLBCL-LT is based on a combination of clinical evaluation, histopathological examination, and immunohistochemical studies. Neoplastic cells exhibit an immunoblastic or centroblastic phenotype and express markers such as CD20, CD79a, and MUM1/IRF4, with frequent positivity for Bcl-2 and Bcl-6. Exclusion of extracutaneous involvement at the time of diagnosis is crucial and requires a complete staging through PET/CT scan and bone marrow biopsy (6).

The standard treatment for PCDLBCL-LT consists of chemotherapy based on anthracyclines combined with the monoclonal antibody rituximab (R-CHOP), with or without local radiotherapy. However, the disease remains associated with a poor prognosis, and ongoing research is evaluating the effectiveness of new therapies, including CAR-T cell therapy or immune checkpoint inhibitors, in patients with refractory or relapsed disease (1, 7)In the absence of proven therapeutic options, relapse/refractory PCDLBCL-LT is generally treated with the same aggressive chemo-immunotherapy regimens used for systemic diffuse large B-cell lymphoma (DLBCL) (8).

Here, we present the case of a man with a relapsed/refractory PCDLBCL-LT who obtained an impressive response with the fourth-line treatment Epcoritamab, a new bispecific antibody targeting anti-CD20.

## 2 Case description

In March 2022, a 72-year-old male patient was referred to our institution presenting with erythematous, non-pruritic, and non-painful nodular lesions on his left leg, which had been present for a few months. A skin biopsy was performed, which confirmed the presence of a diffuse large B-cell lymphoma, non-GCB type, according with Hans alghorithm, with morphology and phenotype consistent with primary cutaneous “leg type” presentation. Immunohistochemical staining (IHC) was positive for CD20, BCL2, BCL6, IgM, and partial c-myc staining (Fluorescence *in situ* hybridization (FISH) confirms the absence of a c-myc traslocation). Tissue specimens showed a lack of immunoreactivity with CD10, MUM1, CD30, and CD5. The proliferative index, evaluated using Ki-67, was 80%. In April 2022, the patient started the first line of treatment with the R-COMP regimen (Rituximab, cyclophosphamide, vincristine, liposomal doxorubicin, prednisone), completing six cycles, and achieving a complete metabolic response by the end of the third cycle. To consolidate the response, the patient also underwent radiotherapy, delivering 32 Gy in 20 daily fractions to the site of the lymphoma lesion on the left leg, completed in December 2022. In February 2023, a Positron Emission Tomography (PET) scan with ^18^F-Fluorodeoxyglucose (FDG) revealed disease relapse, with two contiguous areas of increased uptake in the soft tissues of the left thigh on the medial side, suspicious for lymphoma relapse.

**Figure 1:**
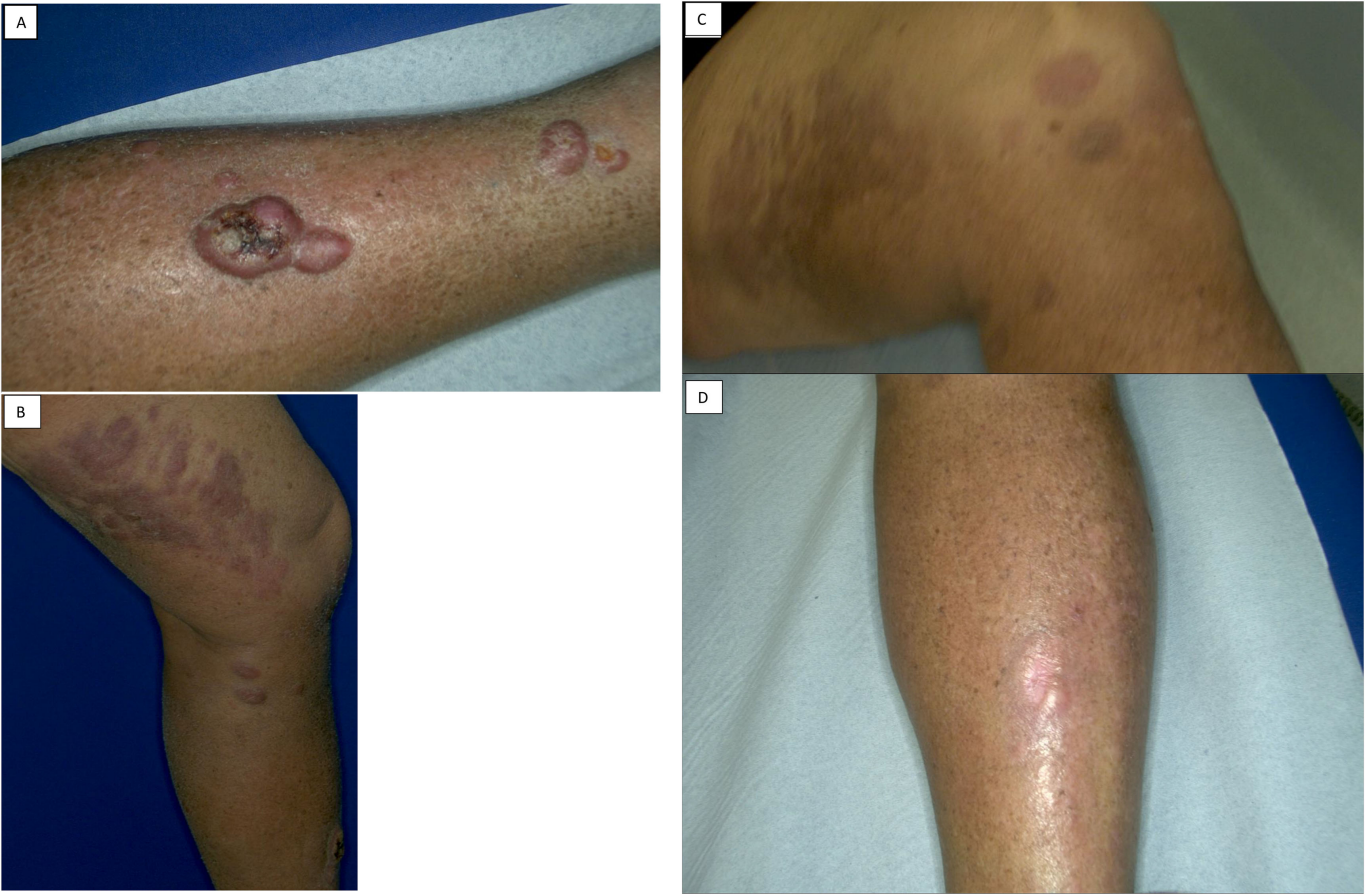
The photos show the lesion in the tibia before **(A, B)** and after 3 cycles with Epcoritamab **(C, D)**.

Considering the patient’s age and comorbidities, including type II diabetes, chronic pancreatitis, arterial hypertension, dyslipidemia, obesity, ischemic heart disease, atheromatosis of the epiaortic vessels, hepatic steatosis, and benign prostatic hyperplasia, a second-line treatment with Tafasitamab combined with Lenalidomide was initiated. The patient completed five cycles of therapy from April 2023 to August 2023, showing complete refractoriness to the treatment and further disease progression documented by PET scan at the end of the fifth cycle. Third-line treatment with Rituximab in combination with Polatuzumab vedotin was then started in September 2023, with a complete response achieved after three cycles and confirmed after six cycles in January 2024 on PET scan. In April 2024, the patient underwent removal of a pre-tibial nodule on the right leg, which histologically showed relapse, with diffuse infiltration of large lymphoid cells, positive for CD20, BCL2, BCL6 and partial MUM1 staining, negative for CD5, CD10, and a proliferative index of Ki67 around 90% (**Figures 1A, B**).

A fourth-line treatment with Epcoritamab, a bispecific antibody targeting CD3 on T cells and CD20 on B cells, was initiated on June 2024. The patient started treatment with a dose escalation regimen beginning with 0.16 mg, followed by 0.8 mg one week later, and ultimately reaching 48 mg after two weeks, showing good tolerance except for a grade 2 cytokine release syndrome (CRS) manifesting initially with shaking chills and fever, and later, after five days, with further fever, moderate hypoxemia (SaO2 = 85%) requiring oxygen support, and mild hypotension. CRS was managed with Tocilizumab 8 mg/kg, resulting in complete symptom resolution within 24 hours.

After the first cycle of Epcoritamab, with two 48 mg doses, the patient demonstrated an excellent response, with regression of cutaneous lesions (**Figures 1C, D**), particularly the regression of the nodules on the right thigh, which were nearly undetectable. The patient said that the local symptoms had completely gone away, he was satisfied with the treatment he had received and carried on taking the drug every week until the end of the third cycle. This meant a total of ten full-dose administrations. After this, the lesions on the right thigh had completely gone. In September 2024, a PET scan showed further disease progression with numerous hypermetabolic lymphoadenopaties involving the right inguinal stations (SUV max 21.2), right external and internal iliac (SUV max=19.1), bilateral common iliac (SUV max 16.2), intra aorto-caval (Suv max = 8.8) and retrocaval (SUV max = 8.8), although a strange complete regression of subcutaneous lesions remained confirmed. It was therefore decided to continue the current therapy with drug administration every two weeks. However, in October 2024, new cutaneous lesions appeared on the abdominal wall and right leg. In November 2024, PET imaging confirmed significant disease progression and a biopsy performed in October from an inguinal lymph node, showed substantial phenotypic changes, with loss of CD20 expression. The phenotypic profile was as follows: CD20 -, CD79a +, Pax5 +, CD30 -, MUM1 +/-, CD10 -, Bcl6 +, Bcl2 +/-, EBV -, MYC translocated. Given the loss of CD20 expression and the disease progression shown by the PET scan, it was decided to start a new line of treatment with Loncastuximab tesirine, an anti-CD19 monoclonal antibody conjugated to a cytotoxic alkylating agent. The patient began this treatment in November 2024, showing initial apparent clinical benefit, with regression of abdominal lymph nodes after the first 150 μg/kg dose. However, after the second cycle (21 days later), swelling of the known lesion on the right leg was observed. Another cycle was administered in January 2025, followed by a PET scan showing further disease progression. The patient was then reassessed for further treatment, but no additional therapy was started due to the patient’s death at the end of January 2025.

## 3 Discussion and conclusion

Primary Cutaneous Diffuse Large B-Cell Lymphoma, Leg Type (PCDLBCL-LT), is a relatively rare disease, accounting for approximately 10–20% of primary cutaneous B-cell lymphoma diagnoses. It predominantly affects elderly patients, typically over 70 years of age, with a higher prevalence in females (3, 4).

The first-line recommended treatment consists of the combination of Rituximab, an anti-CD20 monoclonal antibody, with a multi-agent chemotherapy regimen, the intensity of which depends on the patient’s overall condition, comorbidities, and age. The therapeutic protocols generally follow those used for the treatment of diffuse large B-cell lymphoma (DLBCL) (9).

Patients who are refractory to Rituximab-based chemoimmunotherapy or experience early relapse represent a significant therapeutic challenge, as there are no established second-line treatment options with proven efficacy, taking into account the ineligibility for CAR-T cell therapy and, due to age and comorbidities, the ineligibility for an autologous transplant. Furthermore, due to the rarity of the disease, clinical studies assessing second-line treatment efficacy are lacking. The only exception is a study conducted by a French research group, published in 2017, which reported modest efficacy of single-agent Lenalidomide, with an objective response rate of 26.3% and a progression-free survival (PFS) of 4.86 months (10). Other case reports concerning patients with relapsed/refractory PCDLBCL-LT have also been published. A case series involving two patients has reported the efficacy of treatment with ibrutinib, lenalidomide and rituximab. In both cases, the patients received this triplet at the time of their first relapse, following treatment with R-CHOP. Both patients showed a complete response to the disease, with a duration of response of more than nine months (11). Two other case reports documented the response obtained with ibrutinib. The first one in an elderly patient who had undergone three previous rituximab based lines of therapy. For this 84 year old patient, a complete response disease lasting more than eight months was recorded. The patient finally died from causes unrelated to lymphoma (12). The second case involved a young patient who underwent autologous transplantation after two previous lines of therapy including rituximab. They subsequently relapsed and were treated with lenalidomide. After a further relapse, they were treated with ibrutinib and achieved a complete response that lasted for approximately two years (13). Finally, an abstract documenting an excellent response to single-agent Venetoclax in a 77-year-old patient who had previously undergone chemo-immunotherapy was also published. The patient received the drug at the first relapse and achieved a complete response lasting more than 18 months (14).

The present case report describes the therapeutic choices and responses observed in an elderly, heavily treated patient with a relapsed/refractory PCDLBCL-LT. Notably, this could represent the first documented case of a patient treated with Epcoritamab, a novel bispecific antibody capable of simultaneously binding CD3 on T lymphocytes and CD20 on malignant B cells and activating a T-cell-mediated immune response against lymphoma cells, without previous other immunotherapies.

Interestingly, in this case, Epcoritamab induced a rapid clinical response after the second 48 mg dose; however, an early relapse subsequently occurred, likely due to loss of target antigen expression. This phenomenon has already been described as a potent mechanism of resistance to anti-CD 20 bispecifics (15, 16) and might have been induced by prior extensive exposure to anti-CD20 during previous therapies. However, it is more likely that this resistance is due to the strong selective pressure induced by Epcoritamab, potentially leading to a biological evolution with a progressive downregulation of CD20 expression.

Moreover, it is interesting to underline that initially the disease progressed to other extracutaneous sites, as if the clonal evolution had occurred in a lymph node part of the lymphoma, overcoming the cutaneous tropism and developing only in those sites, while the response remained at the level of the primary cutaneous site. To confirm this hypothesis, it would have been interesting to use a liquid biopsy to evaluate the dynamic changes of circulating tumor DNA. However, this technique is not yet widely used in routine clinical practice. According to the latest evidence collected in the review recently published by Almasri et al. (17), this technique could facilitate the early detection of potential disease progression in cases of B-cell and T-cell lymphomas. In our case, even more interestingly, it would enable us to capture the heterogeneity of lymphoma that may have led to different responses to therapy in different locations, as we observed. This would allow us to better comprehend clonal evolution.

It would have been interesting to try, whether anticipating the use of this drug at earlier lines, and combining it with the use of polychemotherapy regimens, to overcome possible mechanism of clonal evolution or resistance, as already seems to be demonstrated by various published studies (18, 19), would result in a better response in terms of treatment remission.

As this is a description of a single case, it is clear that what has been observed cannot be considered repeatable in other similar cases in the absence of controlled clinical studies documenting this. However reporting outcomes of novel therapies, even in single cases, is of significant interest, particularly when no clinical trials support treatment strategies for relapsed/refractory PCDLBCL-LT. Innovative therapeutic approaches exploiting previously unexplored mechanisms of action may provide new insights into the management of this challenging disease.

## Data Availability

The original contributions presented in the study are included in the article/supplementary material. Further inquiries can be directed to the corresponding author.
